# Antioxidant and Biological Activity of Mexican Madroño Fruit (*Arbutus arizonica*)

**DOI:** 10.3390/foods13182982

**Published:** 2024-09-20

**Authors:** Imelda N. Monroy-García, Pilar Carranza-Rosales, Irma Edith Carranza-Torres, Lelie Denisse Castro-Ochoa, Vianey González-Villasana, Alma Rosa Islas-Rubio, Ezequiel Viveros-Valdez

**Affiliations:** 1 Departamento de Ingeniería Química y Bioquímica, Tecnológico Nacional de México, Instituto Tecnológico de Los Mochis, Juan de Dios Bátiz y 20 de Noviembre, Los Mochis 81259, Sinaloa, Mexico; imelda.mg@mochis.tecnm.mx (I.N.M.-G.); lelie.co@mochis.tecnm.mx (L.D.C.-O.); 2 Coordinacion de Tecnología de Alimentos de Origen Vegetal, Centro de Investigación en Alimentación y Desarrollo, A.C. Gustavo Enrique Astiazaran Rosas #46, Hermosillo 83304, Sonora, Mexico; aislas@ciad.mx; 3 Centro de Investigación Biomédica de Noreste, Instituto Mexicano del Seguro Social, Calle Jesús Dionisio González #501, Monterrey 64720, Nuevo León, Mexico; carranza60@yahoo.com.mx (P.C.-R.); irma.carranzatr@uanl.edu.mx (I.E.C.-T.); 4 Departamento de Química, Facultad de Ciencias Biológicas, Universidad Autónoma de Nuevo León, Av. Pedro de Alba S/N, San Nicolás de los Garza 66450, Nuevo León, Mexico; vianey.gonzalezvl@uanl.edu.mx

**Keywords:** antioxidant activity, biological activity, polyphenols, antiproliferative, wild fruits, *Arbutus arizonica*

## Abstract

The fruit of the Mexican madroño (*Arbutus arizonica*) has been consumed since pre-Columbian times by North American tribes and native groups in Mexico. Despite this, reports on its chemical composition and biological activity are limited. This work aims to determine the antioxidant, antiproliferative, and digestive enzyme inhibition activities of the methanol amberlite-retained extract of Mexican madroño. Results showed that madroño fruit is rich in antioxidants: DPPH (EC_50_ = 0.89 ± 0.03 mg/mL), TEAC (1078 ± 4.9 μM/g), and hemolysis inhibition (IC_50_ = 358.07 μg/mL), with high phenolic and flavonoid content at 15.92 ± 3.2 mg GAE/g and 4.33 ± 0.3 mg CA/g, respectively. Using analytical chromatography, gallic acid, vanillic acid, chlorogenic acid, ferulic acid, quercetin, and rutin were quantified. The extract also showed α-glucosidase inhibition (IC_50_ = 3.1 ± 0.17 mg/mL), but no inhibition against α-amylase and lipase (>5 mg/mL), while showing antiproliferative activity against HeLa, HT-29, and MCF-7 cancer cell lines. These results point towards an interesting potential for the fruit of the *A. arizonica* as chemopreventive and hold potential for elaborating functional foods.

## 1. Introduction

Mexico possesses a wide range of ecosystems and microclimates, featuring diverse vegetation enriched with endemic species and an exceptionally high level of genetic diversity [1]. These species provide an extensive range of plants with fruits, which have biological activities of great interest for the research and development of nutraceutical products [2]. The distinct genotype and formative environment lead to the production of unique and abundant metabolites in wild fruits, providing health benefits. These include vitamins, minerals, and notably large quantities of phytochemicals like polyphenols, flavonoids, and anthocyanins, which exhibit high antioxidant activity. These compounds are related to health benefits, including improved cardiovascular health, enhanced immune function, reduced risk of chronic diseases (e.g., diabetes, cancer), and better cognitive function [3]. In Mexico, edible wild fruits account for 5% of the total species of commercial interest in the country, where 712 species have been recorded [4]. During certain periods, especially in times when other food products cannot be obtained, wild fruits constitute essential nutrient resources. In some communities, the incorporation of wild fruits into the diet contributes to a well-rounded nutritional profile and encourages the conservation of biodiversity, as it promotes the sustainable management of natural habitats and ecosystems where these fruits grow [5].

*Arbutus* L. is a genus that belongs to the Arbutoideae subfamily, a member of the Ericaceae family. The *Arbutus arizonica* tree can reach up to 14 m in height. The fruits are blackish-red berries with a diameter of 6.5–9 mm and contain seeds of approximately 2 mm. Flowering occurs between the months of May and August and fruits from August to October [6]. This species is distributed within the southwest of the United States and northwest of Mexico, mainly in the states of Arizona, New Mexico, Chihuahua, Durango, Jalisco, Sinaloa, Sonora, and Zacatecas [7]. Fruits of the *A. arizonica* have been consumed since pre-Columbian times by North American tribes (Sargent) and continue to be consumed today by native groups of Mexico [8]. In America, another five species of arbutus have been found: *Arbutus madrensis* (Mexico), *Arbutus menziesii* (USA), *Arbutus occidentalis* (Mexico), *Arbutus tessellata* (Mexico), and *Arbutus xalapensis*, (Mexico, Arizona, and Texas), the latter being the most investigated [9]. The fruit and leaf extracts of *A. xalapensis* have shown antioxidant and antibacterial activity against *S. aureus*, *S. tiphymurum*, *L. monocytogenes*, and *E. coli* [10,11]. Also, the leaves are used for the treatment of diabetes in Mexican traditional medicine [12]. *Arbutus unedo* is the most common species in the Mediterranean area and has demonstrated numerous biological activities, such as antidiabetic, antiseptic, diuretic, laxative, anti-aggregant, antimicrobial, and anti-inflammatory effects, among others [9,13,14,15,16]. However, there is little information about the biological activities and chemical composition of most species of *Arbutus*. 

In this work, we report for the first time the total phenolic and flavonoid content and their profile, as well as several biological activities including antioxidant, anti-proliferative, anti-diabetic, and anti-obesogenic activities from the Mexican madroño fruit. The information obtained can be useful for incorporating it into the diet, potentially increasing the intake of bioactive compounds to improve population health, or for the design of nutraceuticals and added value products.

## 2. Materials and Methods

### 2.1. Plant Material and Phytochemical Extraction

Fruits of the *A. arizonica* were collected at Chipinque hill in the city of San Pedro Garza García, México (25.6063° N, 100.3721° W). The plant was identified to species level by the botanic department at FCB-UANL. For the extraction, whole fruits (150 g) were washed and ground in a blender with distilled water (1:5 *w*/*v*). Later, the juice was filtered and passed through an Amberlite XAD-7 column and eluted with methanol. The extract was concentrated in a rotary evaporator (Yamato Model RE2000, Beijing, CN), giving 0.9752 g with a yield of 0.65%, and kept in sealed amber bottles under refrigerated conditions until use [17]. All chemicals used in the different analysis were purchased from Sigma-Aldrich (St. Louis, MO, USA).

### 2.2. Determination of Total Phenolic Content (TPC)

The total phenolic content was determined using the method proposed by Singleton et al., using a Folin–Ciocalteu reagent [18] with slight changes. An amount of 30 μL of madroño extract, 150 μL of Folin–Ciocalteu (1:10), and 120 μL of 7.5% of sodium carbonate were mixed in a 96-well plate and incubated under dark conditions at 25 °C. The absorbance was measured at 764 nm after 30 min using gallic acid as a standard. The results were expressed as milligrams of gallic acid per gram of dry extract. 

### 2.3. Determination of Total Flavonoids Content (TFC)

The total flavonoid content was evaluated using the aluminum chloride assay [19]. An aliquot of 250 μL of the extract or catechin as standard (50–500 mg/L) were mixed with 75 mL of 7% sodium carbonate and 1 mL of deionized water. Five minutes later, at room temperature, 75 mL of 10% (*w*/*v*) AlCl_3_ solution was added. After one minute, 500 μL of 1 M NaOH and 600 µL of deionized water were added and strongly shaken. The absorbance was measured at 496 nm against the blank. The data obtained were expressed as milligrams of catechin equivalents per gram of dry extract. 

### 2.4. Determination of the Phytochemical Composition by RP-HPLC

Phytochemical profiles were assessed according to the method reported previously by Cervantes et al. [20]. The experimental measurements were performed on an RP-HPLC (Agilent Technologies, Walnut Creek, CA, USA) coupled to a photodiode array detector (DAD) using a C18 110A column with a size particle of 5 μm. The fruit extract (1 mg/mL) was separated through a mobile phase consisting of acetonitrile (A) and 0.1% formic acid solution (B). The elution system was from 0 to 35 min in 8% of A and 92% of B under the following conditions: column temperature, 24 °C; flow rate, 1 mL/min; and injection volume, 10 µL. Phenolic compounds were identified at 320 nm by comparing retention times with their respective standards and quantified using calibration curves.

### 2.5. Antioxidants Assays

#### 2.5.1. 2,2′-Diphenyl-1-picrylhydrazyl (DPPH) Assay

The method used to stabilize the 1,1-diphenyl-2-picrylhydrazyl radical (DPPH) was proposed by Braca et al., with slight modifications [21]. A total of 10 mL of 1 mM DPPH stock solution in methanol was prepared, stirred until complete solubilization, and stored at 4 °C protected from light. From this stock solution, 50 mL of 150 μM DPPH methanol was prepared. For the inhibition of the DPPH radical, madroño extracts were added to a 150 µM methanol solution of DPPH in a 1:1 ratio. The absorbance was measured using a UV–Vis spectrophotometer at a wavelength of 517 nm after 30 min of incubation under dark conditions. The percentage of antioxidant activity (AA) was calculated with the following formula:

AA% = 100 − [(Abs sample − Abs blank) × 100/Abs control]

A standard curve was prepared with Trolox and compared the antioxidant activity with the sample.

#### 2.5.2. 2,2′-Azino-bis-3-ethylbenzothiazoline-6-sulfonic Acid (ABTS) Assay

The method followed was proposed by Viveros-Valdez et al., with slight modifications [22]. The radical was obtained after the reaction of ABTS•+ (7 mM) and potassium persulfate (2.45 mM), incubated at room temperature and in darkness for 16 h. The solution was diluted with methanol until we obtained an absorbance of 0.70 (±0.02) at 734 nm. For the assay, 20 μL of madroño extracts were placed in a 96-well plate with 200 µL of the ABTS•+ radical solution for 20 min under dark conditions. Then, the absorbance was measured at 734 nm. A calibration curve was prepared with the synthetic antioxidant Trolox. The results are expressed in mM Trolox equivalents (T.E.) per gram of dry extract.

The changes in the absorbance of the reagent with respect to the blank were used to calculate the antioxidant capacity using the following equation:

AA% = 100 − [(Abs sample − Abs blank) × 100/Abs control]

#### 2.5.3. Protective Effect of Hemolysis Induced on Human Erythrocytes

The methodology used to induce hemolysis using an AAPH (2-2′-Azobis (2-methyl propionamidine dihydrochloride) radical was proposed by Silva-Beltrán et al. [23]. The samples were obtained from healthy volunteers following the reading and signing of informed consent. An amount of 5 mL of heparinized peripheral blood was obtained by venipuncture, which was centrifuged at 1500 rpm for 10 min at room temperature to separate the plasma and leukocytes from the erythrocytes. The erythrocyte pellet was washed three times with 5 volumes of phosphate-buffered saline (PBS) with pH 7.4 and centrifuged at 2500 rpm for 10 min. The erythrocytes were resuspended in 4 volumes of PBS solution until reaching a density of 8 × 10^9^ cells/mL. The AAPH radical triggers the oxidation of membrane lipids and proteins, leading to the hemolysis of erythrocytes. The erythrocyte suspension was mixed with madroño extracts (500, 750, 1000, 2000 µg/mL) and 300 mM of AAPH was dissolved in the PBS (1:1:1 V/V/V). 

The reaction mixture was subjected to gentle shaking during a 3 h incubation at 37 °C. Following incubation, the mixture was diluted with eight volumes of PBS and centrifuged at 4000 rpm for 5 min. The absorbance of the supernatant was measured at 540 nm using a microplate reader. The percentage inhibition was determined using the following equation:

% Inhibition = [AAAPH − AExtract]/[AAAPH × 100]

where AAPH represents the absorbance of AAPH at 540 nm and AExtract represents the absorbance of the extracts at the same wavelength. The concentration of extract required to achieve 50% inhibition (IC_50_) was determined from the dose-response curve, which was constructed by plotting the percentage of hemolysis inhibition against the extract concentration. These calculations were based on data from three independent experiments.

### 2.6. Inhibition of Digestive Enzymes

#### 2.6.1. Inhibition of α-Glucosidase

The α-glucosidase inhibitory activity was assessed using a modified version of the chromogenic method proposed by Kaskoos [24]. The enzyme α-glucosidase (0.8 U/mL) was incubated at 37 °C for 15 min. Then, 50 µL of each fruit extract and 50 µL of the enzyme were combined in a 96-well plate and incubated at 37 °C for an additional 15 min. Following this, 50 µL of a 625 mM *p*-nitrophenyl-α-D-glucopyranoside (PNPG) solution was added to each well and the mixture was incubated for another 15 min. The reaction was stopped by adding 100 µL of 0.2 M Na_2_CO_3_, and the absorbance was measured at 405 nm using a UV–visible microplate reader. Acarbose was used as a positive control and the IC_50_ values were calculated using probit analysis.

#### 2.6.2. Inhibition of α-Amylase

The α-amylase inhibitory activity was assessed following the method described by Sudha et al., with modifications [25]. After pre-incubating the α-amylase (1 U/mL) at 37 °C for 30 min, 50 µL of fruit extract with the same volume of the enzyme were incubated in a 96-well plate at 37 °C for 15 min. Subsequently, 50 µL of a 0.5% starch solution in phosphate buffer (pH 6.9) was added to each well, and the reaction was further incubated for 20 min at 37 °C. The reaction was terminated by adding 20 µL of 1 M HCl, followed by the addition of 50 µL of iodine reagent (I_2_ 3 mM and KI 30 mM). The absorbance was read at 750 nm using a UV–visible microplate reader. Acarbose was used as a positive control.

#### 2.6.3. Inhibition of Pancreatic Lipase

The inhibitory activity against pancreatic lipase was evaluated based on the method described by Maqsood et al., with some modifications [26]. Pancreatic lipase (2.5 mg/mL) was prepared in a phosphate buffer solution (PBS, 60 mM, pH 8). The reaction mixture consisted of 100 µL of serial dilutions of the fruit extract or Orlistat^®^ combined with 30 µL of the lipase solution, incubated at 37 °C for 15 min. Subsequently, 10 µL of the substrate p-NPP (10 mM in DMSO) was added. During the reaction, the lipase enzyme hydrolyzes p-NPP, releasing p-nitrophenol, a yellow-colored product that can be quantified at 405 nm. After an additional incubation of 30 min at 37 °C, the absorbance was read using a microplate reader.

The percent inhibition for the three enzymes was calculated using the following formula:

% Inhibition = [Ac − As/Ac] × 100

where Ac and As represent the absorbance of the control and the sample, respectively. The control contained all components except the test sample. Orlistat and acarbose provided by Sigma-Aldrich (St. Louis, MO, USA) served as positive controls. The IC_50_ values were determined through logarithmic regression analysis.

### 2.7. Antiproliferative Assay

The inhibitory effect on cell proliferation of the madroño fruit was assessed using a previously described method by Viveros-Valdez et al. [27]. Three cancer cell lines, HeLa (human cervical carcinoma ATCC^®^ CCL-2), MCF-7 (human breast cancer ATCC^®^ HTB-22), and HT-29 (human colorectal adenocarcinoma ATCC^®^ HTB-38), were provided by Dra. Carranza-Rosales from Centro de Investigación Biomédica del Noreste/Instituto Mexicano del Seguro Social (CIBIN/IMSS). These cells were initially cultured in 25 cm^2^ tissue culture flasks with Dulbecco’s modified eagle medium (DMEM) supplemented with Ham’s nutrients (Ham’s F-12) and 10% fetal bovine serum (FBS) (pH = 7.2). An antibiotic mixture of penicillin and streptomycin (10,000 IU/mL: 10,000 µg/mL; 1 mL of mixture/1 L of medium) was added to the culture medium. The cells were maintained in an incubator at 37 °C with a 5% CO_2_/95% air atmosphere at constant humidity. Cell density was determined by trypsinization, and viability was assessed using Trypan Blue (4%).

For the evaluation of the antiproliferative activity, 5000 cells of each cancer cell line were seeded in a 96-well plate. After 24 h of incubation, 100 µL of madroño extract at concentrations of 125, 500, 750, 1000, and 2000 µg/mL were added and further incubated for 24 h. Subsequently, 20 µL of a 10% Alamar Blue Invitrogen^®^ (Carlsbad, CA, USA) solution was added to each well. The plate was incubated with agitation and fluorescence was measured 4 h later using a fluorometer (FLx800 Bio-Tek Instruments, Inc., Winooski, VT, USA) with an excitation wavelength of 535 nm and emission at a wavelength of 595 nm. Taxol was utilized as a positive control. The IC_50_ values were determined through probit analysis.

## 3. Results and Discussion

The *Arbutus* genus has received great interest as it represents a promising source of healthy phytochemicals. Various compounds with biological activities have been reported from different *Arbutus* species; however, the beneficial effects are mainly related to the presence of phenolic compounds [9]. For this reason, incorporating them into a diet could provide health benefits, as it is known that fruits of Mediterranean *Arbutus* species have been used in traditional medicine and the development of functional foods [28,29]. Moreover, wild edible fruits, including Mexican madroño, are a significant source of food and nutrition in some local communities, contributing to improved nutrition and food security in Mexico [8]. Therefore, recognizing the biological value of fruits from *Arbutus arizonica*, they can be used for the production of innovative functional foods, bringing economic benefits.

### 3.1. Total Phenolic Content (TPC) and Total Flavonoid Content

The number of phenolic compounds present in the fruit is determined by the extraction process, the genotype, and the environmental conditions [30]. Polyphenols are known to play an important role as antioxidants in human nutrition and health [31]. Members of the Arbutus genus are generally recognized for their high phenolic content, which includes both flavonoids and phenolic acids, contributing to their antioxidant activities [32]. The total phenolic content of the wild Mexican madroño was 15.92 ± 3.2 mg GAE/g of extract (Table 1).

This result is similar to that previously reported by Doukani and Tabak [33] and Isbilir et al. [34] for fruit extracts of Algerian and Turkish Arbutus unedo (7.02 to 14.74 mg GAE/g and 14.29 mg GAE/g, respectively) and higher than that of the *Arbutus andrachne* extract where the total phenols ranged from 2.42 to 4.1 mg GAE/g [35]. Oliveira et al. [36] evaluated the influence of the ripening stage on the total phenol content of *Arbutus unedo* fruits and their results showed almost double the amount of TPC in the unripe and intermediate stage of ripeness. This study was carried out with ripe fruits, so immature fruits probably contain more total phenols. Nevertheless, the wild Mexican madroño could be considered as a good source of polyphenols with high antioxidant potential.

Flavonoids are a group of polyphenolic compounds found in many plants, including Arbutus species, that offer various health benefits and are responsible for many of the colors in fruits. Flavonoids have been reported to exhibit various biological activities, including antioxidant, anti-inflammatory, anti-cancer, anti-diabetic, and anti-microbial effects [37]. The total flavonoid content of the Mexican madroño fruit was 4.33 ± 0.3 mg CA/g of extract. The amount of flavonoids was higher than that found in bitter orange (3.83 ± 0.05 RU/g of extract), litchi (3.8 ± 2.2 mg QE/g of extract), and madroño (3.2 mg QE/g of extract) [38,39,40]. 

### 3.2. Phenolic Acids and Flavonoids by HPLC-DAD

**Figure 1:**
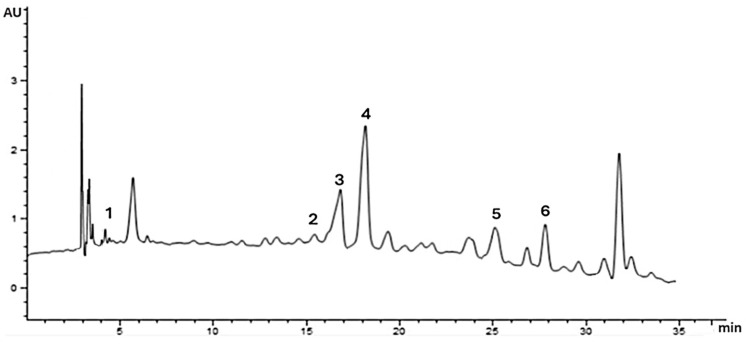
HPLC chromatogram from methanol amberlite-retained extract of *A. arizonica*. 1: Gallic acid, 2: Chlorogenic acid, 3: Vanillic acid, 4: Ferulic acid, 5: Rutin, and 6: Quercetin.

Phenolic acids exhibit various biological activities, including antioxidant and anticancer properties [41]. The content of free phenolic acids and flavonoids in Mexican madroño fruit are shown in Table 2. Chromatographic analysis (Figure 1) revealed the presence of four phenolic acids belonging to the hydroxybenzoic acid subclass (Gallic and vanillic) and hydroxycinnamic acids (ferulic and chlorogenic), and two flavonoids (quercetin and rutin). Regarding phenolic acids, vanillic acid was the most abundant with value of 3.38 mg/g, followed by ferulic acid (2.44 ± 0.02 mg/g) and gallic acid (0.55 ± 0.005 mg/g), being chlorogenic acid with the lower amount (0.46 mg/g of extract). The amount of phenolic acids in *Arbutus unedo* fruit was evaluated by Ayaz et al. and, unlike our results, gallic acid was the main phenolic acid, with 10.7 ± 0.04 mg/g of extract and vanillic acid in lower quantities (0.12 ± 0.08 mg/g of extract) [42]. Similar amounts of vanillic acid were found in *Amburana cearinsis* (2.7 mg/g of extract), showing a decrement in pro-inflammatory cytokine levels in rats, mainly attributed to the vanillic acid content [43]. Nevertheless, vanillic acid also has demonstrated antioxidant, antiproliferative, antimicrobial, and neuroprotective activities [44,45,46,47]. 

Regarding ferulic acid, the ethanol fraction of Spiranthes sinensis, a famous herb used in traditional Chinese medicine, was 2.87 mg/g of extract, practically the same amount found in the Mexican madroño [48]. Ferulic acid is recognized for possessing various biological activities, including antioxidant, anti-inflammatory, antiviral, antiallergic, antibacterial, antithrombotic, anticancer, and hepatoprotective effects [49,50]. Otherwise, rutin was the main flavonoid found in Mexican madroño extract with 2.28 mg/g of extract, containing more than grapefruit (1.39 mg/g of extract), while lemon and shamouti orange contained larger quantities (7.21 and 6.56 mg/g of extract, respectively) [51]. Rutin has demonstrated important biological activities such as antioxidant, anti-inflammatory, cardioprotective, anticancer, neuroprotective, antimicrobial, and hepatoprotective effects [52,53,54,55,56,57].

### 3.3. Antioxidant Capacity

The antioxidant capacity was measured by in vitro assays using DPPH● and ABTS•+ radicals (Table 1). The DPPH● antiradical activities of the Mexican madroño showed an EC_50_ = 0.89 ± 0.03 mg/mL. The wild fruits *E. tinifolia* and *S. lanuginosum* showed a higher antioxidant activity (EC_50_ = 0.32 ± 0.03 mg/mL and 0.48 ± 0.05 mg/mL, respectively) [17]. However, pulp and peel extracts from *Cydonia oblonga* fruit showed similar DPPH● free radical scavenging activities (EC_50_ of 0.6 and 0.8 mg/mL, respectively) [58]. However, the Mexican madroño showed a higher activity than the XAD7 extract of the *Prumnopitys andina*, with an EC_50_ of 0.93 ± 0.03 mg/mL [59]. On the other hand, in the ABTS•+ assay, the Mexican madroño showed an antioxidant capacity value equivalent to Trolox (CAET), at 1078 ± 4.9 μM/g, presenting a greater antiradical activity compared to other wild fruits such as *Prunus espinosa* (5080 μM/g), *Rubus ulmifolius* (4810 μM/g), and *Arbutus unedo* (4480 μM/g) [60]. 

The importance of conducting multiple assays of antioxidant capacity lies in the fact that different antioxidants exhibit variable reactivity towards free radicals depending on their chemical structure and functional groups. By using assays at multiple wavelengths, the diverse antioxidant capacities of compounds present in a sample can be captured, providing a more comprehensive assessment of its antioxidant potential. The ABTS assay primarily measures the ability of antioxidants to scavenge the ABTS•+ radical cation, while the DPPH assay assesses their ability to neutralize the DPPH• radical. Since these radicals have distinct chemical properties, the evaluation of antioxidant capacity using both assays allows us to understand how different compounds perform against different types of radicals, enhancing the robustness of antioxidant characterization [61].

Erythrocyte cells are constantly exposed to oxidative stress due to their role in oxygen transport, making them highly susceptible to oxidative damage. Therefore, assessing the antioxidant capacity of natural products using erythrocytes provides a biologically relevant model that reflects the interaction between antioxidants and oxidative stress in living organisms [62]. For this assay, AAPH was used as a generator of peroxyl radicals to induce hemolysis in human erythrocytes. The protective capacity of the Mexican madroño on red blood cells yielded an IC_50_ value of 358.07 μg/mL (Table 1). Compared with other fruit extracts, such as *Cydonia oblonga* (IC_50_ = 652 μg/mL), *Piracanta coccinea* (IC_50_ = 451.34 μg/mL), *Condalia hookeri* (IC_50_ = 899.06 μg/mL), and *Mangifera indica* (520 μg/mL produced a 35% inhibition of hemolysis), extracts of *A. arizonica* presented a greater antioxidant capacity in an ex vivo model [17,58,63]. Assessing the antioxidant capacity of Mexican madroño extract in erythrocytes allows for the observation of real-time responses to oxidative stressors, providing insights into the cellular adaptive mechanisms elicited by the phenolic compounds [64]. These results offer a physiologically relevant and dynamic approach that can provide valuable discoveries into their potential health benefits and therapeutic applications.

### 3.4. Digestive Enzymatic Ihibition

At present, only a limited number of α-glucosidase inhibitor drugs, including acarbose and voglibose, have received approval for the treatment of diabetes. The chemical structures of these drugs primarily consist of sugar moieties [65]. In addition, undesirable side effects have been observed with the administration of these medications, such as abdominal distention, flatulence, and diarrhea [66]. As a result, numerous studies have concentrated on identifying alternative α-glucosidase inhibitors with non-sugar core structures, particularly polyphenols, given their widespread natural occurrence and potential biological activities [67]. In the α-glucosidase assay, Mexican madroño extract presented inhibitory activity with an IC_50_ value of 3.1 ± 0.17 mg/mL, which is greater than that found in fruits such as *Prunus persica* (IC_50_ = 10.9 ± 0.23 mg/mL), *Vactinium cyanococcus* (IC_50_ = 13 mg/mL), and *Prunus salicina* (IC_50_= 6.06 ± 0.02 mg/mL) [68,69]. However, under the same experimental conditions, acarbose (positive control) presented a higher inhibition of α-glucosidase with an IC_50_ value of 0.13 mg/mL. Enzyme inhibition is typically associated with the relationship between structure and activity. The number of -OH substituents on phenolics acids and flavonoids increase the inhibition activity [70].

The enzyme α-amylase is one of the key enzymes in the human digestive system, which degrades starch to monosaccharides and causes an increase in blood glucose [71]. Natural amylase inhibitors offer an attractive therapeutic approach for the treatment of postprandial hyperglycemia by decreasing glucose released from starch [72]. The Mexican madroño extract showed a low α-amylase activity, with IC_50_ values > 5 mg/mL. Despite this, the extract was able to inhibit 45.12% of the enzyme at a concentration of 5 mg/mL (Table 2). In other studies, extracts rich in polyphenols from different types of berries inhibited α-amylase in vitro, and the most effective were raspberry and rowanberry (IC_50_ of 21 and 4.5 μg/mL, respectively). They observed that the degree of α-amylase inhibition was related to appreciable amounts of soluble tannins [73].

A crucial objective in the quest to combat obesity is to discover compounds that can hinder the digestion and absorption of nutrients. One promising strategy to tackle obesity involves inhibiting pancreatic lipase (PL) to reduce the absorption of lipids [74]. Mexican madroño extracts do not show inhibitory activity against pancreatic lipase (IC_50_ > 5 mg/mL). McDougall et al. evaluated the inhibition activity of lipase enzymes from different berries extracts. Blackcurrant and rowan extracts were not able to inhibit lipase, blueberry extract showed slight inhibition, with lingonberry, arctic bramble, cloudberry, strawberry and raspberry capable of considerably inhibiting lipase activity [75]. The authors suggest that inhibition may be related to the presence of ellagotannins. The fruit extracts evaluated in this research are low in tannin content, which may explain the low inhibitory activity of α-amylase and lipase.

### 3.5. Antiproliferative Activity

Wild fruits with high phenolic content have been shown to be powerful antioxidants for the prevention on certain types of cancer [76]. Mexican madroño extracts showed dose-dependent antiproliferative activity toward MCF-7, Hela, and HT-29 cells. The highest antiproliferative activity of the extract was exhibited on HT-29 cells with an IC_50_ = 1.786 ± 0.12 mg/mL, followed by MCF-7 cells with an IC_50_ = 2.070 ± 0.24 mg/mL, with HeLa cells being the weakest (IC_50_ = 3.319 ± 0.37 mg/mL) (Table 3). These IC_50_ values are lower than those reported by Popović et al. for several fruits of the genus Prunus, which exhibit antiproliferative activity toward HT-29 with IC_50_ values > 4.79 ± 0.14 mg/mL [77] and EC_50_ values > 6.68 ± 0.29 mg/mL [78]. The antiproliferative activity of Mexican madroño extract may be attributed to its high content of phenols (vanillic and ferulic acids) and flavonoids (rutin), compounds which have been reported to possess anticancer properties [45,49,50,52,53,54]. It has been proposed that the anticancer activity of phenolic compounds such as ferulic acid and vanillic acid can be attributed to specific mechanisms. Ferulic acid may exert its effects through pro-apoptotic activity, enhancing the expression and protein levels of caspases and Bax, and modulating p53 [79]. Similarly, vanillic acid has been reported in in vivo models to reduce carcinogenesis by restoring antioxidant levels and enzymes involved in xenobiotic metabolism, thereby promoting anti-inflammatory and protective activities [80]. Likewise, various studies have demonstrated that flavonoids such as rutin induce cellular apoptosis through the upregulation of miRNA-877-3p expression and the secretion of tumor necrosis factor-alpha (TNF-α) in various tumor cell types [81,82].

## 4. Conclusions

Fruit from the Mexican madroño (*Arbutus arizonica*) exhibits significant antioxidant activity. This is the first report where the antiproliferative effect against cancer cell lines and glucosidase enzyme inhibition are demonstrated, highlighting its potential as a source of bioactive compounds. The rich phenolic and flavonoid content, along with the presence of key bioactive compounds such as vanillic and ferulic acids, as well as the flavonoids quercetin and rutin, suggests that the fruit of the Mexican madroño could be valuable in the development of functional foods and chemopreventive agents. These findings support further research into the possible health benefits of this wild fruit.

## Figures and Tables

**Table 1:** Total phenolic content (TPC), total flavonoid content, and antioxidant capacity of *Arbutus arizonica* extract.

Fruit	TPC [mg EAG/g] *DW	TFC [mg EAG/g] *DW	TEAC [μM/g] *DW	DPPH EC_50_ [mg/mL]	Hemolysis Inhibition IC_50_ [µg/mL]
*Arbutus arizonica*	15.92 ± 3.2	4.33 ± 0.3	1078 ± 4.9	0.89 ± 0.12 ^a^	358.07 ± 47 ^a^
** Control	—	—	—	0.014 ± 3 ^b^	290 ± 30 ^a^

* DW: dry weight. Values are the average ± standard deviation of three parallel experiments. ** Trolox was used on DPPH free radical scavenging assay and ascorbic acid on AAPH-induced hemolysis. *n* = 3, different letters in the same column indicate significant differences (*p* < 0.05).

**Table 2:** Content of phenolic acids and flavonoids by HPLC-DAD of *A. arizonica* extract.

Compound	Rt (min)	Content * mg/g	Ecuation	R^2^
Gallic acid	4.41	0.553 ± 0.056	y = 255.41x + 106.72	0.9999
Chlorogenic acid	15.54	0.459 ± 0.003	y = 217.64x − 97.638	0.9965
Vanillic acid	16.44	3.380 ± 0.061	y = 727.99x − 2451.8	0.9972
Ferulic acid	18.12	2.441 ± 0.024	y = 464.55x − 127.53	0.9898
Rutin	25.37	2.289 ± 0.066	y = 89.555x + 94.519	0.9991
Quercetin	28.15	1.674 ± 0.029	y = 87.583x − 107.99	0.9989

* Values represent the samples mean ± SD (standard deviation, *n* = 3).

**Table 3:** Inhibition of digestive enzymes and antiproliferative activity of *A. arizonica* extract.

Fruit	Half Maximal Inhibitory Concentration [mg/mL]						
	α-Glu	α-Amy	Lipase	MCF-7	HeLa	HT-29	* RBCs
*A. arizonica*	3.1 ± 0.2 ^a^	>5	>5	2.07 ± 0.3 ^a^	3.32 ± 0.4 ^a^	1.79 ± 0.1 ^a^	>5
** Control	0.15 ± 0.04 ^b^	1.07 ± 0.1	0.21 ± 0.05	0.016 ± 0.003 ^b^	0.014 ± 0.005 ^b^	0.017 ± 0.002 ^b^	ND

* RBCs = Red blood cells = Erythrocytes. ** Acarbose was used on α-glucosidase and α-amylase assays, orlistat on lipase inhibition, and Taxol against cancer cells lines, *n* = 3. Different letters in the same column indicate significant differences (*p* < 0.05).

## Data Availability

The original contributions generated for this study are included in the article; the data presented in this study are available on request from the corresponding author.
